# Differences in Coma Recovery Scale-Revised performance in an upright position versus lying position

**DOI:** 10.3389/fneur.2025.1683275

**Published:** 2025-10-07

**Authors:** Andrew DaCosta, Aya Bou Fakhreddine, Stephanie Stroever, Ryan Stork, Katherine O’Brien, Bei Zhang

**Affiliations:** ^1^Disorders of Consciousness Rehabilitation Program, TIRR Memorial Hermann Hospital, Houston, TX, United States; ^2^LPG Memory Care, Lee Health System Inc, Fort Myers, FL, United States; ^3^Department of Physical Medicine and Rehabilitation, The University of Texas Health Science Center at Houston John P and Katherine G McGovern Medical School, Houston, TX, United States; ^4^The College of Public Health and Social Justice, Saint Louis University, St. Louis, MO, United States; ^5^Department of Medical Education, Texas Tech University Health Sciences Center, Lubbock, TX, United States; ^6^Division of Physical Medicine and Rehabilitation, Department of Neurology, Texas Tech University Health Sciences Center, Lubbock, TX, United States

**Keywords:** disorders of consciousness, traumatic brain injury, Coma Recovery Scale-Revised, neurological rehabilitation, acquired brain injury

## Abstract

The study investigated the impact of patient positioning on behavioral assessment of consciousness in individuals with a disorder of consciousness (DoC) using the Coma Recovery Scale-Revised (CRS-R). In this retrospective study, 1,470 CRS-R assessments were performed on 232 patients in four different positions: lying in bed (Bed), sitting at edge of mat (Mat), sitting in a wheelchair (Wheelchair), and standing (Standing), in an acute inpatient rehabilitation setting. A conditional random coefficients multi-level model was used to examine changes in the transformed CRS–R total unit (which converted the raw CRS-R total score to an equal-interval scale) across positions, accounting for repeated measurements within subjects and variability introduced by different raters. Transformed CRS–R total unit was significantly associated with assessment position. Compared to the Bed position (controlling age, gender, etiology, number of arousal protocol used, and days post-injury), patients assessed in the Wheelchair, Mat, and Standing positions had estimated 2.7-, 3.2-, and 3.5-unit increases in the transformed CRS–R total unit (*p* = 0.02, 0.01, and 0.11), respectively. Number of arousal protocols used was not significantly associated with assessment position. Increased use of these protocols did not enhance CRS–R performance. Improved physical and cognitive functionality in an upright position, rather than arousal alone, may contribute to the improvements on the CRS-R. Our results revealed that patients scored higher on the CRS-R in an upright position compared to a lying position. This suggests that the CRS-R is better performed in an upright position instead of a lying position in patients with DoC. We recommend assessing the level of consciousness in patients with DoC in an upright position and out of bed whenever feasible.

## Introduction

Disorders of consciousness (DoC) are severe alterations in level of arousal and awareness following an extensive injury to the brain (1). Common causes include traumatic brain injury, hypoxic–ischemic brain injury, and stroke (1). Clinically, consciousness is defined as the state of awareness of the self and environment (2). Adequate arousal (wakefulness) is a prerequisite to revealing one’s level of awareness (2). DoC states are classified as coma (lack of arousal and awareness), unresponsive wakefulness syndrome (UWS; presence of arousal but lack of awareness), and minimally conscious state (presence of arousal with fluctuating but reproducible signs of awareness) (3). More recently, minimally conscious state (MCS) has been divided into MCS- and MCS + based on the absence or presence of linguistic processing (4). Given that the level of consciousness concerns a varying degree of awareness and cognitive functions, which has to be supported by sufficient arousal, it is prudent to eliminate variables that negatively impact one’s level of arousal and awareness during the clinical assessment.

Previous studies have shown how verticalization influences arousal and functional performance (5). Bringing patients with DoC into an upright position to optimize their level of arousal recovery is a commonly employed strategy in DoC rehabilitation programs (5). It is widely accepted that verticalization (i.e., being in an upright position, which could be sitting or standing with a standing frame or weight reduction harness system) can be introduced in the early stages of rehabilitation following a severe brain injury to prevent the deleterious effect of immobility on the cardiovascular system and other bedridden complications (6). In the real world, adopting verticalization to optimize arousal and reveal signs of consciousness has been more commonly applied based on practical experience but less well-studied (5). Evidence has shown that tilt tables can improve the level of consciousness (7, 8). It is highly recommended to use the well-studied standardized scale, Coma Recovery Scale-Revised (CRS-R), at least 5 times within 2 weeks to optimize the diagnostic accuracy (9). However, its reliability and accuracy may still be affected by the assessment setup (10), with the patient’s position being a key factor.

## Methods

This study aims to assess the impact of patient’s position on the assessment of consciousness in patients with DoC. This was achieved by retrospectively analyzing a large pool of CRS-R total scores recorded in 4 positions, i.e., lying in bed (Bed), sitting at the edge of a mat (Mat), sitting in a wheelchair (Wheelchair), and up in a standing position (Standing), at the discretion of the evaluators when assessing and treating patients with DoC based on real-world clinical needs in an acute inpatient rehabilitation unit. We hypothesized that the patients would perform better, or achieve higher scores, on the CRS-R when in an upright position (i.e., Mat or Wheelchair or Standing) than in a lying position (i.e., Bed).

This is a retrospective chart review study from a single institution cohort. The cohort contains 232 patients who were consecutively admitted to a specialized acute inpatient rehabilitation program with a diagnosis of DoC from 2020 to 2023 that underwent 1,470 assessments of level of consciousness using the JFK CRS-R (11). Demographic information and clinically relevant variables were extracted from the electronic medical record (EMR) for review. The study was approved by the local institutional review board.

### Setting

A pre-admission screening was conducted to determine the appropriateness of admission to the program. Admissions criteria include the presence of a DoC and the patient’s medical status being stable for the logistical transfer to the facility. The program accepts all patients presented with DoC, regardless of whether they need ventilation support (12).

### Inclusion and exclusion criteria

All patients participated in at least 3 h of rehabilitation therapy, daily, 5 days a week. These included therapies provided by well-trained physical therapists, occupational therapists, speech language pathologists, and neuropsychologists specializing in brain injury and DoC. Rehabilitation goals aim at identifying signs of consciousness, facilitating the emergence of consciousness, and improving neuromuscular status and cardiopulmonary conditioning, etc. (12).

### Primary outcome measure

**Figure 1:**
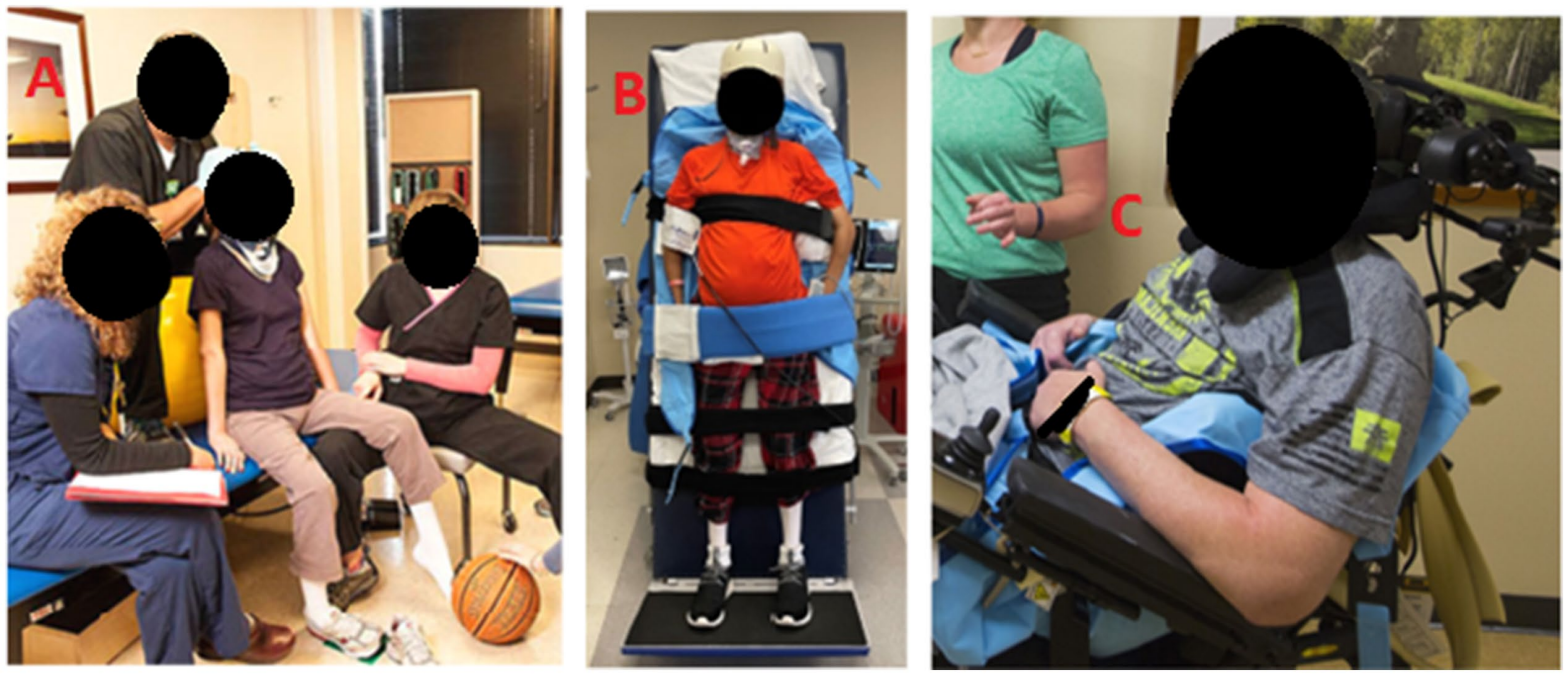
**(A)** Demonstrates the assessment position of Mat. **(B)** Demonstrates the assessment position of Standing. **(C)** Demonstrates the assessment position of Wheelchair.

The CRS-R is one of the most commonly used behavioral proxies for assessing the level of consciousness (11, 13). It is comprised of 6 hierarchical subscales that assess auditory, visual, motor, oromotor, communication, and arousal functioning. Items within each subscale are designed to measure reflexive behaviors to cognitively mediated behaviors. Scoring on the CRS-R ranges from 0 to 23, with higher scores representing higher levels of consciousness (11). The arousal facilitation protocol in the user manual provides specific instructions on the location, intensity, and maneuver of applying deep pressure to a patient’s body parts to prolong their arousal status (14). The CRS-R was administered twice weekly as appropriate. Patients were assessed by the dyads of a neuropsychologist and occupational therapist or a speech language pathologist and physical therapist. All therapists were trained based on the CRS-R user manual and has passed a competency assessment to ensure standardization of instrument. Patient’s position at the time of testing was dictated by real-world clinical and logistical needs, e.g., time allotted for the assessment, tolerance to transfer due to neuromuscular complications, availability of space and equipment, and other treatment needs. Specific examples could include a patient may have been assessed in their wheelchair if they were scheduled to participate in therapies either before or after the assessment, or a patient may have been assessed in bed if there was not adequate time to transfer the patient to an upright position and still complete the full assessment in the allotted time within their therapy schedule. Patients were assessed in one of four positions: lying in bed (Bed), sitting at the edge of a mat (Mat), sitting in a wheelchair (Wheelchair), and up in a standing position (Standing). Examples of these positions can be seen in Figure 1. The Bed position involved the patient laying in a supine position with the head of the bed raised to approximately 30 degrees. The Mat position involved the patient seated upright at the edge of an elevated therapy mat. Trunk support was provided by an additional therapy tech who sits behind the patient if a patient was unable to support themselves. In the Wheelchair position, patients were seated upright in either a power wheelchair or tilt-in-space wheelchair. The Standing position involved the patient being upright either in a tilt table or a standing frame or a body weight supported gait training system. After each assessment, the CRS-R total score, subscores, patient’s position at the time of CRS-R, command/object used, number of arousal facilitation protocol (AFP) used per session, and the assessors were documented in a built-in template in the EMR system.

### Data elements, retrieval, and conversion

Basic demographic information (age and gender), injury characteristics (etiology), and admission DoC diagnosis was retrieved from the EMR. The CRS-R total score, days post-injury when the CRS-R was performed (calculated by using the date difference between the CRS-R and the injury), patient’s position at the time of CRS-R, and the number of arousal protocols used per session were also obtained from each patient and each assessment. Injury characteristics were coded as traumatic, hypoxic ischemic, cerebrovascular or other based on information from their rehabilitation admission documentation. Positioning was characterized as Bed, Mat, Wheelchair, or Standing based on where the patient was oriented at the time of each CRS-R performed. The above data was retrieved from all the 1,470 CRS-Rs performed for the 232 patients. The CRS-R total raw scores were converted to 0–100 units based on the transformed CRS-R Rasch person measure proposed by Weaver et al. and Weaver et al. (15, 16), which makes it an equal-interval scale and provides better calibration for analysis, comparison, and interpretation of the CRS-R total scores. In short, the term “transformed CRS-R total unit” was utilized in the following context to distinguish it from the raw CRS-R total score.

### Statistical analysis

All data management and analyses were conducted using STATA statistical software version 17.0 by experienced statisticians. We examined the distribution of variables by conducting descriptive analysis. This included frequency and percentage for categorical variables and mean and standard deviation for continuous variables. Normality of the continuous outcomes was assessed using Shapiro–Wilk test. To address the hierarchical structure of the data (multiple assessments within each subject) and to examine the changes of transformed CRS-R total units based on the positions, a conditional random coefficients model was used. This multi-level model accounted for the clustering of repeated measurements within subjects and the variability introduced by different raters. The model was adjusted for gender, age, cause of brain injury, number of arousal protocols used per session, and days post injury at which CRS-R was performed. These variables were controlled for as there is evidence that they can contribute to recovery outcomes (17). Moreover, the association between the number of arousal protocols and position was assessed separately using negative binomial mixed effect model to account for the multiple measurements per subject and different raters. For all hypothesis testing, statistical significance was determined at an alpha level of 0.05.

## Results

### Participants

Demographic and clinical characteristics are summarized in Table 1. Mean age was 37.4 years with a standard deviation (SD) of 16.2 years. Most of the included patients were males (65.1%). The most common cause of injury was traumatic brain injury accounting for 47.0% of cases followed by hypoxic–ischemic brain injury (26.7%), and cerebrovascular injury (19.0%). On average each patient underwent 10.3 (SD = 6.8) assessments via the CRS-R. These assessments were done on average of 89.8 (SD = 5.9) days post-injury. The mean CRS-R total score per subject was 7.4 (SD = 4.1) out of 23. The mean transformed CRS-R total unit per subject was 40.4 (SD = 11.8) out of 100. A post-hoc simulation-based power analysis using the observed repeated-measures structure indicated that the study had approximately 86% power to detect a small (e.g., 1.5 point) difference in CRS-R scores between positions at *α* = 0.05.

**Table 1:** Basic information of the cohort (*N* = 232).

Demographic item	Data
Age (years)	37.4 ± 16.2^*^
Gender (*N*)	
Female	81 (34.9%)
Male	151 (65.1%)
Cause of brain injury	
Traumatic (TBI)	109 (47.0%)
Hypoxic Ischemic (HIBI)	62 (26.7%)
Cerebrovascular	44 (19.0%)
Other^a^	17 (7.3%)
Days post-injury when the CRS-R was administered	89.8 ± 5.9^*^
Assessment number per subject	10.3 ± 6.8^*^
CRS-R total score per subject	7.4 ± 4.1^*^
Transformed CRS-R total unit per subject	40.4 ± 11.8*
CRS-R total score per position	
Bed	10.6 ± 5.1*
Mat	12.8 ± 4.5*
Standing	13.1 ± 4.4*
Wheelchair	12.3 ± 4.7*
Number of arousal protocols used per subject	3.0 ± 2.1^*^

*N*, number; CRS-R, Coma Recovery Scale-Revised.

^*^ Mean ± Standard Deviation.

a Other includes: encephalopathy (metabolic or toxic), combination of dual causes, or other causes.

### Transformed CRS-R total units by positions

Table 2 shows the associations between assessment positions and transformed CRS-R total units. The transformed CRS-R total unit was significantly positively associated with the Mat, Wheelchair, or Standing positions, when taking the Bed position as the reference after controlling for gender, age, cause of brain injury, number of arousal protocols, and days post injury at which CRS-R was assessed. Patients assessed in the Mat position had an estimated 3.2-unit increase in the transformed CRS-R total unit compared to those in the Bed position (*p* = 0.01). Similarly, those assessed in the Wheelchair position had almost an estimated 2.7-unit increase in the transformed CRS-R total unit compared to those in the Bed position (*p* = 0.02). Patients assessed in the Standing position demonstrated an estimated 3.5-unit increase in the transformed CRS-R total unit compared to those in the Bed position, although not statistically significant (*p* = 0.11). These results suggested a patient’s better performance was achieved in the Mat, Wheelchair, and Standing positions in the real-world clinical setting when using the CRS-R to assess the level of consciousness. Collectively, when combining all the upright body positions, that is Mat+Wheelchair+Standing, the transformed CRS-R total unit was significantly positively associated with the upright position. Patients had an estimated 2.9-unit increase in the transformed CRS-R total unit compared to those in the Bed position (*p* = 0.01), suggesting an overall better performance in the upright positions.

**Table 2:** Association between positions and transformed CRS-R total units.

	Coefficient (95% CI)	*p* value
Position during assessment^a^^,^^b^		
Mat	3.2 (0.9–5.4)	**0.01**
Wheelchair	2.7 (0.5–4.9)	**0.02**
Standing	3.5 (−0.8–7.8)	0.11
Upright^c^	2.9 (0.8–5.0)	**0.01**
Age	−0.1 (−0.2 – −0.04)	**0.00**
Gender		
Male	1.2 (−1.1–3.5)	0.31
Cause of brain injury^d^		
HIBI	−2.7 (−5.3–0.04)	0.05
CVA	1.4 (−1.9–4.7)	0.42
Other^e^	−4.2 (−8.4–0.04)	0.05
Non-traumatic^f^	−1.8 (−4.2–0.6)	0.13
Days post-injury of CRS-R		0.32
Number of arousal protocols used	−2.0 (−2.4 – −1.7)	**0.00**

CRS-R, Coma Recovery Scale-Revised; CI, confidence interval; HIBI, hypoxic–ischemic brain injury; CVA, cerebrovascular accidents.

a Using multi-level mixed effect model and taking the Bed position as the reference.

b Adjusted for adjusted for gender, age, cause of brain injury, number of arousal protocols used per session, and days post injury at which CRS-R was performed.

c Combining the data of Mat, Standing, and Wheelchair.

d Taking traumatic brain injury as the reference.

e Other includes: encephalopathy (metabolic or toxic), combination of dual causes, or other causes.

f Combining the data of HIBI, CVA, and Other.

**Figure 2:**
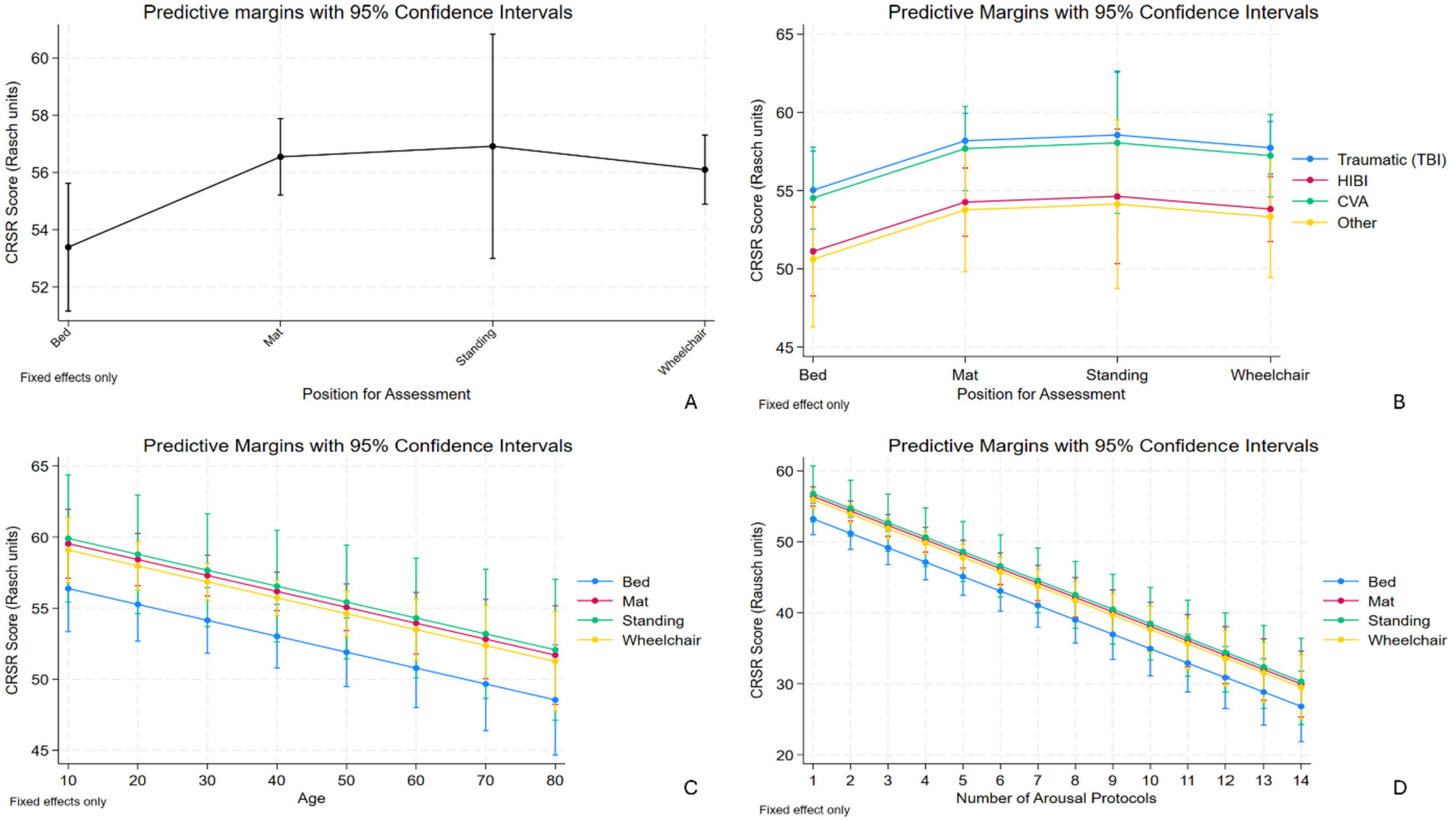
**(A)** Demonstrates the predictive margins of transformed CRS-R total units in the upright positions being significantly higher than those in Bed position. **(B)** Demonstrates the predictive margins of transformed CRS-R total units in different positions plotted over the cause of brain injury. **(C)** Demonstrates the predictive margins of transformed CRS-R total units in different positions plotted over the age. As the age increases, the transformed CRS-R total unit decreases, with the lowest being in Bed. **(D)** Demonstrates the predictive margins of transformed CRS-R total units in different positions plotted over the number of arousal facilitation protocol (AFP) used. As the number of AFP increases, the transformed CRS-R total unit decreases, with the lowest being in Bed. CRS-R, Coma Recovery Scale-Revised; CI, confidence interval; HIBI, hypoxic–ischemic brain injury; CVA, cerebrovascular accidents.

The transformed CRS-R total unit was also found significantly negatively correlated with age, when controlling for the abovementioned confounders (*p* < 0.01). As the age increases by 10 years, the transformed CRS-R total unit decreases by 1.1-unit, suggesting that older patients tend to have a lower score on the CRS-R. Additionally, the transformed CRS-R total unit was not significantly correlated with the cause of brain injury when comparing the traumatic and non-traumatic causes. Otherwise, the transformed CRS-R total unit was not significantly associated with gender and days post-injury. The predictive margins of transformed CRS-R total units in different positions and those plotted over the causes of brain injury and age were presented in Figures 2A–C.

### Number of AFP used by positions

Interestingly, the transformed CRS-R total unit was found significantly negatively correlated with the number of AFP used per session. For each one unit increase in the number of AFP used, the transformed CRS-R total unit decreased by 2.0-unit (*p* < 0.01; Table 2) when controlling for the confounders, suggesting that patients who received more AFP also had lower CRS-R total scores. The predictive margins of transformed CRS-R total units in different positions plotted over the number of AFP used were presented in Figure 2D. However, the number of AFP used per session is not statistically correlated with assessment positions, taking the Bed position as the reference, with or without controlling the confounders (Table 3). Otherwise, the number of AFP used was significantly positively correlated with age, while not with gender, cause of brain injury, and days post-injury (Table 3).

**Table 3:** Association between positions and numbers of arousal facilitation protocols used.

	Coefficient (95% CI)	*p* value
Position during assessment^a^^,^^b^		
Mat (unadjusted)	−0.3 (−0.7–0.2)	0.22
Mat (adjusted)	−0.3 (−0.7–0.1)	0.19
Wheelchair (unadjusted)	−0.08 (−0.5–0.3)	0.69
Wheelchair (adjusted)	−0.09 (−0.5–0.3)	0.62
Standing (unadjusted)	−0.6 (−1.5–0.2)	0.13
Standing (adjusted)	−0.7 (−1.5–0.1)	0.11
Age	0.01 (0.01–0.2)	**0.001**
Gender		
Male	−0.02 (−0.3–0.3)	0.89
Cause of brain injury^c^		
HIBI	0.2 (−0.1–0.5)	0.17
CVA	−0.1 (−0.5–0.3)	0.61
Other^d^	0.2 (−0.3–0.7)	0.35
Days post-injury of CRS-R		0.08

CI, confidence interval; HIBI, hypoxic–ischemic brain injury; CVA, cerebrovascular accidents; CRS-R, Coma Recovery Scale-Revised.

a Using multi-level mixed effect model and taking the Bed position as the reference.

b Adjusted for adjusted for gender, age, cause of brain injury, and days post injury at which CRS-R was performed.

c Taking traumatic brain injury as the reference.

d Other includes: encephalopathy (metabolic or toxic), combination of dual causes, or other causes.

## Discussion

The results demonstrate that patients with DoC performed better on the CRS-R when assessed in an upright position as opposed to in a lying position. These findings are consistent with the previous literature regarding verticalization and level of consciousness (5, 7). Optimizing the assessment position is particularly relevant given the well-documented concern of misdiagnosis of DoC (18). Approximately 40% of patients diagnosed as being in a state of unresponsive wakefulness syndrome were actually in a minimally conscious state (18). Accurate assessment of the level of consciousness is crucial as it influences patient care trajectories (19).

The ascending reticular activating system (ARAS) plays a critical role in arousal and consciousness (20). Vestibular system input is hypothesized to affect functioning of the ARAS (21). This underlying mechanism would be consistent with our findings that the performance of patients with DoC during the standardized assessment using CRS-R improves in vertical positions. This includes all three different types of verticalization: sitting at the edge of a mat, sitting in a wheelchair, and being in a standing position however it is achieved. The former two involve the head/neck and upper body verticalization, while the last one involves the head/neck and full body verticalization as well as weight-bearing through the lower extremities. Relatively speaking, maintaining one’s position in a wheelchair is the easiest; maintaining one’s position sitting at the edge of a mat is more challenging and effortful; and maintaining a standing position is the most energy-consuming and potentially painful. Therefore, different upright positions may elicit different levels of underlying physical reactions and neural firing. Sometimes, the standing position (or any upright positions) may be too uncomfortable for the patient to engage and perform in a task. The assessor will need to adjust the position and allow for the best possible performance. The overall stimulating effects might be reflected in the correlation coefficients corresponding to the transformed CRS-R total units in the three upright positions compared to Bed. The conversion has been proved to be able to reflect the true change of CRS-R beyond measurement error, avoid inaccurate inferences of a change from the ordinal scale, and allow for interpretation and comparison of therapeutic effects across studies (16). Our results revealed that with being in Wheelchair, Mat, and Standing positions, it may increase the transformed CRS-R total units by 2.7, 3.2, and 3.5, respectively, and collectively as an upright position by 2.9; all with statistical significance except for Standing. Based on the current understanding, a 4-unit change corresponds to 0.2 standard deviation (SD) of the minimal clinically important differences (MCID) of the transformed Rasch units of CRS-R, indicating a small detectable difference, while a 9-unit change corresponds to 0.5SD MCID and a moderate detectable difference (16). The significance of our results could be manifested by comparing it to the randomized controlled trial of amantadine for severe traumatic brain injury (16, 22). Using the same conversion, the difference of the transformed CRS-R total units between the amantadine and placebo groups at the end of the 4-week trial was 3.6 units, approximating the 0.2SD MCID, but was not statistically significant (16). The comparison demonstrates the average impact of placing a patient in an upright position is relatively comparable to that of giving amantadine over 4 weeks in addition to natural recovery and inpatient rehabilitation; moreover, such an effect has statistical significance (except for Standing). In brief, our results suggest that adopting an upright position (especially sitting at the edge of a mat and in a wheelchair) for the CRS-R assessment would be considered clinically meaningful.

Secondly, the AFP was designed to improve the arousal level of the patient and facilitate their participation and performance during the CRS-R. However, increased indication for use of AFP could suggest worse patient arousal. We found that while the transformed CRS-R total unit was significantly associated with the number of AFP administered, however, the correlation was negative. For each additional use of AFP, the transformed CRS-R total unit was 2.0-unit lower. Additionally, it might be assumed that improved performance on the CRS-R in upright positions was due to enhanced arousal levels, which should have led to a decreased need for AFP in these positions. This effect was not confirmed in our data. The number of AFP administered did not significantly vary by position, despite a small tendency in decreased use of AFP (Wheelchair −0.09 vs. Mat −0.3 vs. Standing −0.7), indicating that its usage was fairly consistent across different assessment positions. These results demonstrate that the increased use of AFP may be attributed to lower arousal levels in certain patients at baseline, which was correlated with poorer CRS-R scores among those patients. The AFP alone was inadequate to significantly improve a patient’s CRS–R performance as intended. Overall, these findings suggest that the assessment position is a more crucial factor in supporting a patient’s CRS-R performance. Improved physical and cognitive functionality in an upright position might be contributing to the improvements on the CRS-R, rather than improved arousal alone. Nonetheless, the AFP should still be applied as indicated, regardless of the assessment position.

Other findings of the study include that age was significantly associated with CRS-R performance, although it may not be clinically significant; as with every 10-year increase in age, the transformed CRS-R total unit decreases only by 1.1-unit. The impact of different upright positions on CRS-R remained statistically significant independent of the effect of age.

The significance of this study lies in its direct relevance to real-world clinical practice and its potential to address the persistent challenge of high misdiagnosis rates. The research aligns with ongoing efforts to identify subtle yet critical factors that may influence patient performance and diagnostic accuracy, even when standardized assessment tools like the CRS-R are employed. Ensuring that patients are evaluated in the most favorable position is crucial for accurately determining their level of consciousness. Investigating different positions and identifying the optimal one for assessment could represent a simple yet impactful step toward improving diagnostic precision. In particular, upright positions deserve heightened attention from clinicians.

### Study limitations

A few limitations of the study are worth mentioning. Patients were not assessed by the same examiners during all the assessments. Scoring biases may exist among examiners despite standardized training. Real-world clinical factors may influence positioning decisions during assessments. For instance, due to the time constraint, a patient may be assessed in bed in order to achieve timely completion of the assessment. Moreover, a physical therapist, given their scope of practice, may be more inclined to assess a patient in a standing position compared to other therapy disciplines. Therefore, the effect of positioning on the assessment outcome was not studied in a controlled manner. Despite these limitations, the study benefits from a large sample size, which ensures sufficient statistical power and enhances the validity of the findings. Otherwise, the current study did not explore if positioning impacts all subscales equally or disproportionately impacts specific subscales, nor did it examine potential changes in diagnostic categories. These warrant further investigation.

## Conclusion

Our results revealed that patients scored significantly higher on the CRS-R in an upright position compared to a lying position. This suggests that the CRS-R is better performed in an upright position instead of a lying position in patients with DoC. We recommend assessing the level of consciousness in patients with DoC in an upright position and out of bed whenever feasible.

## Data Availability

The raw data supporting the conclusions of this article will be made available by the authors, without undue reservation.
